# A clinically significant interaction between voriconazole and rifapentine: a case report and review of evidence

**DOI:** 10.3389/fmed.2026.1761845

**Published:** 2026-01-23

**Authors:** Tingting Chen, Xiaoling Chen, Qingquan Zhang

**Affiliations:** 1 Department of Pharmacy, Quanzhou First Hospital, Quanzhou, Fujian, China; 2 Department of Intensive Care Medicine, Quanzhou First Hospital, Quanzhou, Fujian, China

**Keywords:** drug–drug interaction, rifapentine, therapeutic drug monitoring, tuberculosis, voriconazole

## Abstract

We present a case of concurrent pulmonary aspergillosis and tuberculosis in a 53-year-old male, treated with voriconazole and rifapentine. In this case, co-administration with rifapentine resulted in a markedly lower voriconazole trough concentration (0.4 μg/mL on day 7) compared to that without it (4.3 μg/mL on day 25), reflecting a 90.7% reduction. After rifapentine was discontinued and the voriconazole dose was increased to 300 mg q12h intravenously (day 8), the trough concentration remained at 0.4 μg/mL two days later (day 10). Subsequently, it increased to 3.0 μg/mL by day 14 (6 days post-adjustment) and further rose to 10.8 μg/mL by day 18 (10 days post-adjustment), exceeding the therapeutic range. The results demonstrated a significant decrease in voriconazole levels during combination therapy, an effect that persisted for over one week after rifapentine was discontinued. This case illustrates that increasing the voriconazole dose immediately after rifapentine cessation is not advisable to counteract this interaction. Furthermore, therapeutic drug monitoring should be continued even after target trough levels are attained, as the waning enzyme-induction effect may subsequently lead to supra-therapeutic exposure and potential toxicity.

## Introduction

Voriconazole is a first-line broad-spectrum triazole antifungal agent for *Aspergillus* infections. Its use is complicated by nonlinear pharmacokinetics and substantial inter-individual variability, necessitating therapeutic drug monitoring (TDM) with a target trough concentration of 1–5.5 mg/L (1–3). Moreover, as a substrate and an inhibitor of CYP2C9, CYP2C19, and CYP3A4, voriconazole has a high potential for drug–drug interactions (4). This risk is particularly concerning in patients with tuberculosis, a population at high risk for invasive aspergillosis (5) who may concurrently receive potent CYP450 inducer rifapentine. However, clinical reports documenting this specific interaction remain scarce.

We report a case of pulmonary aspergillosis and tuberculosis co-infection in a 53-year-old man treated with voriconazole and rifapentine. In this case, voriconazole trough concentrations were monitored at six time points over a 19-day period, spanning both the pre- and post-discontinuation phases of rifapentine therapy. The results revealed a significant decrease in voriconazole levels during the combination therapy, which persisted for over one week after rifapentine was discontinued.

## Case

A 53-year-old male patient weighing 45 kg was admitted to our hospital on 20 October 2025, with a chief complaint of a productive cough for over six years, worsened in the past 10 days. Twelve years ago, the patient was diagnosed with pulmonary tuberculosis at another hospital and received a standard 8-month anti-tuberculosis regimen. Six years ago, he was diagnosed with chronic obstructive pulmonary disease (COPD) and has since been regularly using indacaterol/glycopyrronium powder for inhalation and procaterol granules for symptom control. Five months prior to this admission, he presented to our hospital with shortness of breath and was diagnosed with bilateral secondary pulmonary tuberculosis (bacteriologically positive, retreatment) and pulmonary disease due to *Mycobacterium avium-intracellulare* complex (MAC). The patient developed drug-induced liver injury during initial anti-tuberculosis therapy with the HRZE regimen (isoniazid, rifampin, pyrazinamide, and ethambutol), necessitating the replacement of pyrazinamide and rifampin with levofloxacin and rifapentine. Following the identification of a co-existing MAC infection, azithromycin was added to the regimen for the treatment of the nontuberculous mycobacterial (NTM) infection. Upon discharge, he was prescribed and regularly took the following medications: rifapentine (0.45 g, twice weekly), isoniazid (0.3 g, once daily), ethambutol (0.75 g, once daily), levofloxacin (0.5 g, once daily), and azithromycin (0.5 g, once daily). Ten days prior to this admission, the patient experienced worsened cough and sputum production without an obvious trigger. The sputum was thick and difficult to expectorate, accompanied by dyspnea at rest and orthopnea, prompting his return to our hospital.

On admission, vital signs were as follows: temperature 36.5 °C, heart rate 108 beats/min, respiratory rate 25 breaths/min, and blood pressure 98/57 mmHg. Physical examination of the chest revealed diminished breath sounds with concomitant rhonchi and crackles bilaterally. Laboratory Investigations: Arterial blood gas analysis (on room air) revealed severe respiratory acidosis: pH 7.054, PaCO₂ 76.1 mmHg, PaO₂ 112 mmHg, and lactate 4.2 mmol/L. Concurrent electrolyte testing showed severe hyponatremia (sodium 115 mmol/L) and hyperkalemia (potassium 5.1 mmol/L). Complete Blood Count: white blood cell count 9.93 × 10⁹/L, neutrophils 8.12 × 10⁹/L, hemoglobin 147 g/L. Inflammatory Markers: C-reactive protein 43.36 mg/L, procalcitonin 1.174 ng/mL. Renal Function: Serum creatinine 39.2 μmol/L, blood urea nitrogen 3.15 mmol/L. Liver function and biochemistry: Albumin 34.7 g/L, total bilirubin 17.1 μmol/L, aspartate aminotransferase 50 U/L, alanine aminotransferase 22 U/L. Chest CT demonstrated a cavitary lesion in the left upper lobe. Admission Diagnoses: Severe Community-acquired Pneumonia (CAP) with Type II Respiratory Failure, Bilateral Secondary Pulmonary Tuberculosis (Retreatment), Pulmonary Disease due to MAC and COPD.

**Figure 1:**
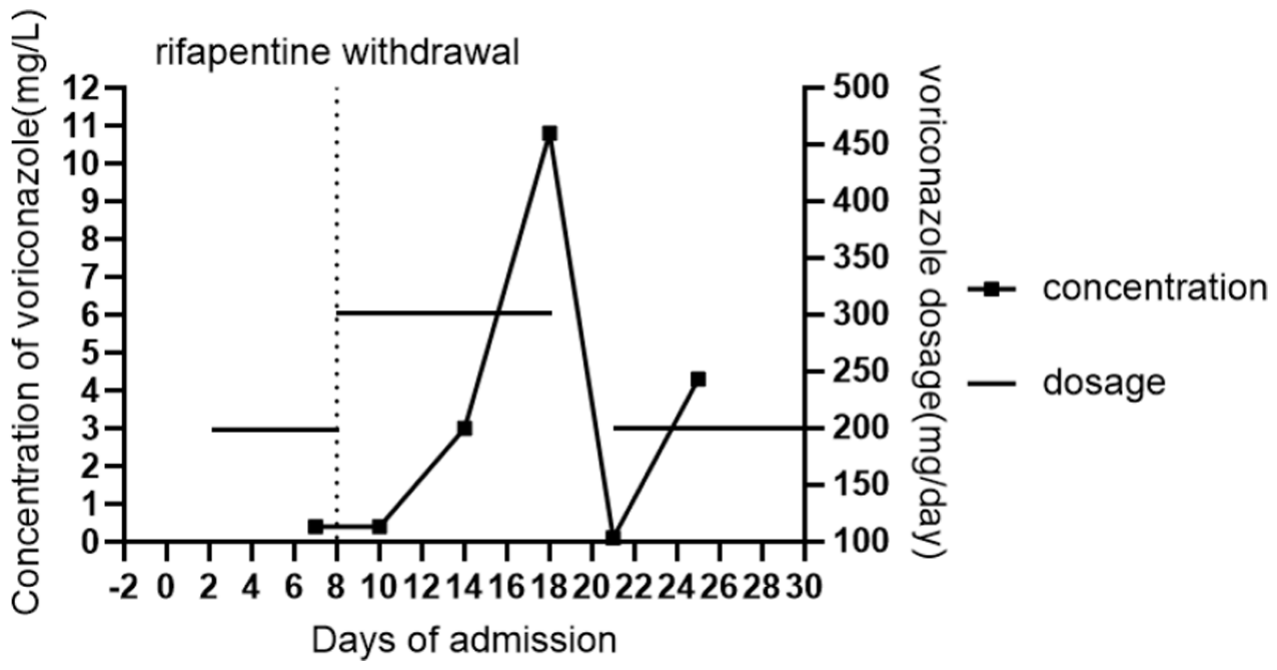
Changes in voriconazole trough concentration in the patient. Day “0” denoted the day of the current admission.

Following admission on October 20, the patient underwent urgent nasotracheal intubation and was initiated on ventilator support (PC-BIPAP mode, rate 15 bpm, FiO₂ 50%, PS 15 cmH₂O, PEEP 5 cmH₂O). Empirical antimicrobial therapy with piperacillin-tazobactam (4.5 g IV q8h) was commenced. The pre-existing anti-mycobacterial regimen was continued, consisting of oral rifapentine (0.45 g twice weekly, Monday and Thursday), isoniazid (0.3 g daily), and ethambutol (0.75 g daily), supplemented with intravenous levofloxacin (0.5 g daily) and azithromycin (0.5 g daily). On October 22, a blood PCR test was positive for *Aspergillus* species (910 copies/mL), prompting the initiation of intravenous voriconazole with a loading dose (300 mg q12h) followed by a maintenance dose (200 mg q12h). The diagnosis of invasive aspergillosis was further supported by positive galactomannan (GM) and 1,3-*β*-D-glucan (G) tests in bronchoalveolar lavage fluid on October 24. Therapeutic drug monitoring of voriconazole revealed a subtherapeutic trough level of 0.4 μg/mL on October 27. Consequently, rifapentine was discontinued on October 28 due to its potential to induce voriconazole metabolism, and the voriconazole dose was increased to 300 mg q12h. However, a repeat measurement on October 30 showed the trough level remained low at 0.4 μg/mL. Subsequent levels eventually rose to 3.00 μg/mL (November 3) and 10.8 μg/mL (November 7). The latter elevated level was associated with poor mental status, leading to the temporary withdrawal of voriconazole. By November 10, the voriconazole trough level had decreased to 0.1 μg/mL, and the drug was re-introduced at 200 mg IV q12h. A level measured on November 14 was 4.3 μg/mL, within the therapeutic range. At this point, the patient’s condition had stabilized. He was alert, breathing via a tracheostomy with high-flow oxygen (FiO₂ 30%, flow 30 L/min), and was transferred to a general ward for continued management. Figure 1 demonstrates the significant reduction in voriconazole trough concentrations during rifapentine co-administration and the prolonged interaction after its discontinuation.

## Discussion

Tuberculosis can cause severe pulmonary structural damage, predisposing patients to a variety of pulmonary diseases, including chronic pulmonary aspergillosis (5). Rifapentine, a rifamycin derivative, is one of the key agents in the treatment of active drug-susceptible tuberculosis (6). Voriconazole, a broad-spectrum triazole antifungal, serving as a first-line therapeutic agent for *Aspergillus* infections. The rifamycins act as inducers of CYP450 enzymes, with rifampin being the most potent, rifapentine having an intermediate effect, and rifabutin exhibiting comparatively weak induction (7). Voriconazole is metabolized mainly by CYP2C19 and, to a lesser extent, by CYP3A4 and CYP2C9. Therefore, concomitant use of a rifamycin can markedly increase the metabolic clearance of voriconazole. The therapeutic range of voriconazole (trough level 1.0–5.5 mg/L) is essential for optimizing efficacy while minimizing toxicity. Coadministration with potent CYP450 enzyme inducers like rifapentine significantly accelerates its metabolism, often resulting in subtherapeutic trough concentrations (<1.0 mg/L) and an increased risk of treatment failure. Currently, research on rifamycin drug interactions has largely focused on rifampin, while data regarding rifapentine remain limited (7–10). In the case report by Ling et al. (11), voriconazole trough concentrations measured on days 3 and 5 after rifapentine discontinuation were both below 1.0 μg/mL. Following a dose adjustment to 200 mg q8h and an additional 5 days of treatment (i.e., day 10 after discontinuation), the trough concentration increased to 1.81 μg/mL. In the study by Lu et al. (12), co-administration with rifapentine resulted in a 71.4 to 81.9% reduction in voriconazole trough concentrations in two patients, compared with the levels measured without rifapentine. In the other eight patients who re-initiated voriconazole at least 6 days after discontinuing rifapentine, the trough concentrations were all within the target range. Using a physiologically based pharmacokinetic (PBPK) model, Sandra Grañana-Castillo et al. evaluated the interaction between rifapentine and rilpivirine, demonstrating that the enzyme-inducing effect of rifapentine on rilpivirine was largely eliminated within two weeks after discontinuation (13). Furthermore, available evidence generally suggests that increasing the dosage of either rilpivirine (13) or bedaquiline (14) is not recommended to counteract the enzyme-inducing effects of rifapentine.

In this case, co-administration with rifapentine resulted in a markedly lower voriconazole trough concentration (0.4 μg/mL on day 7) compared to that without it (4.3 μg/mL on day 25), reflecting a 90.7% reduction. After rifapentine was discontinued and the voriconazole dose was increased to 300 mg q12h intravenously (day 8), the trough concentration remained at 0.4 μg/mL two days later (day 10). Subsequently, it increased to 3.0 μg/mL by day 14 (6 days post-adjustment) and further rose to 10.8 μg/mL by day 18 (10 days post-adjustment), exceeding the therapeutic range. Throughout hospitalization, the patient was not administered any medications known to interfere with voriconazole metabolism, and liver function remained stable. These conditions offer an objective assessment of the induction effect of rifapentine on voriconazole metabolism and its duration. These serial concentration changes confirm that rifapentine significantly induces voriconazole metabolism, and this effect persists for at least one week after discontinuation. Importantly, increasing the voriconazole dose immediately after rifapentine discontinuation is not recommended to compensate for this interaction. In addition, repeat monitoring of trough concentrations should be maintained even after they reach the target range following a dose increase, as the waning induction effect may subsequently lead to supra-therapeutic levels and toxicity.

In addition to drug–drug interactions, voriconazole trough concentrations are influenced by CYP2C19 gene polymorphism (15–17). Although CYP2C19 genotyping was not performed in this patient, the voriconazole trough concentration reached the therapeutic target under standard dosing, indicating a limited effect of CYP2C19 metabolic status on steady-state exposure in this case. However, the patient’s genotype might still have modulated the interaction intensity between voriconazole and rifapentine.

This case demonstrates a marked decrease in voriconazole trough concentration due to co-administration with rifapentine, with the interaction persisting for over a week after discontinuation. It is not advisable to increase the voriconazole dose immediately after rifapentine cessation to counteract this interaction. Moreover, therapeutic drug monitoring must continue even once target trough levels are achieved, as the waning induction effect can lead to supra-therapeutic exposure and toxicity. However, this case report has several limitations. First, the CYP2C19 genotype, which is a known factor influencing voriconazole concentrations, was not assessed. Second, the single-case design inherently limits the generalizability of the findings. Future studies should include and analyze multiple similar cases to strengthen the persuasiveness and robustness of the conclusions.

## Data Availability

The original contributions presented in the study are included in the article/supplementary material, further inquiries can be directed to the corresponding author.
